# Ophthalmic artery arising from the external carotid artery system: the middle meningeal artery in South African patients

**DOI:** 10.1186/s12886-023-02996-0

**Published:** 2023-05-29

**Authors:** B.R Omotoso, R Harrichandparsad, L Lazarus

**Affiliations:** 1 Discipline of Clinical Anatomy, School of Laboratory Medicine and Medical Sciences, College of Health Sciences, University of KwaZulu-Natal, Westville Campus, Private Bag X54001, Durban, 4000 South Africa; 2 Department of Neurosurgery, School of Clinical Medicine, College of Health Sciences, Nelson R Mandela School of Medicine, University of KwaZulu-Natal, Durban, South Africa

**Keywords:** Ophthalmic artery, Middle meningeal artery, Retinoblastoma, Digital subtraction angiography, Arteriovenous malformations

## Abstract

**Background:**

The ophthalmic artery is the first branch of the internal carotid artery. It arises from the supraclinoid segment of the internal carotid artery within the subarachnoid space and enters the orbit via the optic canal. However, due to complex embryogenesis, the ophthalmic artery can arise from different parts of the internal carotid artery or the distal branches of the external carotid artery. This is usually associated with a variation in the course of the ophthalmic artery through the superior orbital fissure instead of coursing through the optic canal. The ophthalmic artery and its branches vascularise the eyeball and its contents. Consequently, information about its morphologic variation is essential for treating clinical conditions such as central retinal artery occlusion, retinoblastoma chemoembolization, and ophthalmic artery aneurysm.

**Case presentation:**

We report on two cases of the ophthalmic artery arising from the middle meningeal artery in one adult (33-year-old Indian female) and one pediatric (2-year-old African male) South African patient examined by digital subtraction angiography. The patients were diagnosed with arteriovenous malformations and bilateral retinoblastoma, respectively.

**Conclusions:**

The ophthalmic artery plays a vital role in vision generation. Thus, its anatomy is of clinical interest to neurosurgeons, ophthalmologists, and interventional radiologists.

## Background

The ophthalmic artery (OA) is the first branch of the internal carotid artery (ICA). It arises from the supraclinoid segment of the ICA within the subarachnoid space and enters the orbit via the optic canal [1]. Occasionally, in the presence of anatomical variations, the OA may arise from various segments of the ICA and its branches, including the cerebral arteries [2]. The OA rarely arises from the distal branches of the external carotid artery such as the middle meningeal artery (MMA) [3], and even more rarely from the basilar artery and the posterior communicating artery [2]. The overall prevalence of variation in the origin of the OA is approximately between 2—4% [4, 5], while the reported incidence of variation in origin from the MMA ranges from 1.4—2.5% [2, 5]. Variation in origin of the OA has been associated with some cerebral vascular diseases such as aneurysms, vertebral artery dissection, cerebral infarction, moyamoya disease, arteriovenous malformation, and cavernous malformation, and other non-vascular abnormalities [5, 6]. Atypical origin of the OA from the MMA has been linked with visual complications following surgical intervention around the skull base [7]. Iatrogenic injury or occlusion of the OA due to morphologic variation can result in blindness after surgical intervention around the sphenoid ridge or embolization in the territory of the external carotid artery [1, 8]. We report on incidental findings of right OA arising from the MMA using digital subtraction angiography (DSA). The aim/purpose of this study is to describe a rare variation in the origin and course of the OA in an adult and a pediatric South African patient.

## Case presentation

The design of this study was approved by our Institutional Review Board/Ethics Committee (Biomedical Research Ethics Committee of the University of KwaZulu-Natal with ethical No: BE 148/19). No identifying patient information is present in this paper.

### Case 1

**Fig. 1:**
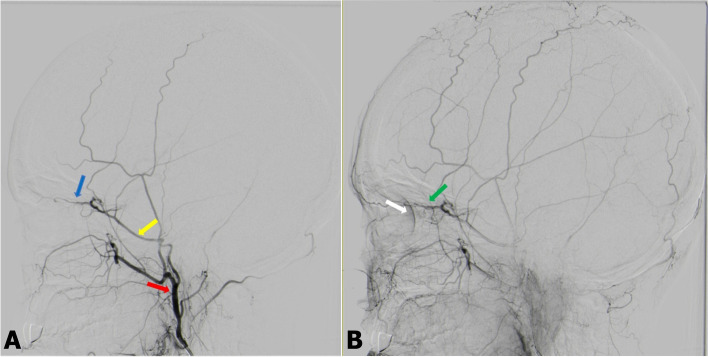
**A** and **B** Digital subtraction angiography of the right external carotid artery in early (**A**) and late arterial phases show a meningo-ophthalmic variant with choroidal blush seen in the late phase (**B**) (Case 1). **A** The red arrow illustrates the right external carotid artery. The yellow arrow illustrates the right middle meningeal artery. The blue arrow illustrates the meningo-ophthalmic connection to the ophthalmic artery. **B** The green arrow illustrates the ophthalmic artery, late phase showing choroidal blush (White arrow)

A 33-year-old female Indian South African patient presented to the hospital with adult-onset of seizures. The patient was admitted for DSA for suspected arteriovenous malformation. The right ICA run displayed filling of a small (< 3 cm) arteriovenous malformation by branches of the middle cerebral artery. There was no filling of the OA from the ICA run. The right external carotid artery (ECA) run illustrated the OA filling from MMA and entering the orbit via the superior orbital fissure (SOF) (Fig. 1A and B). The left OA had a standard origin from the supraclinoid part of the ICA and coursed through the optic canal.

### Case 2

**Fig. 2:**
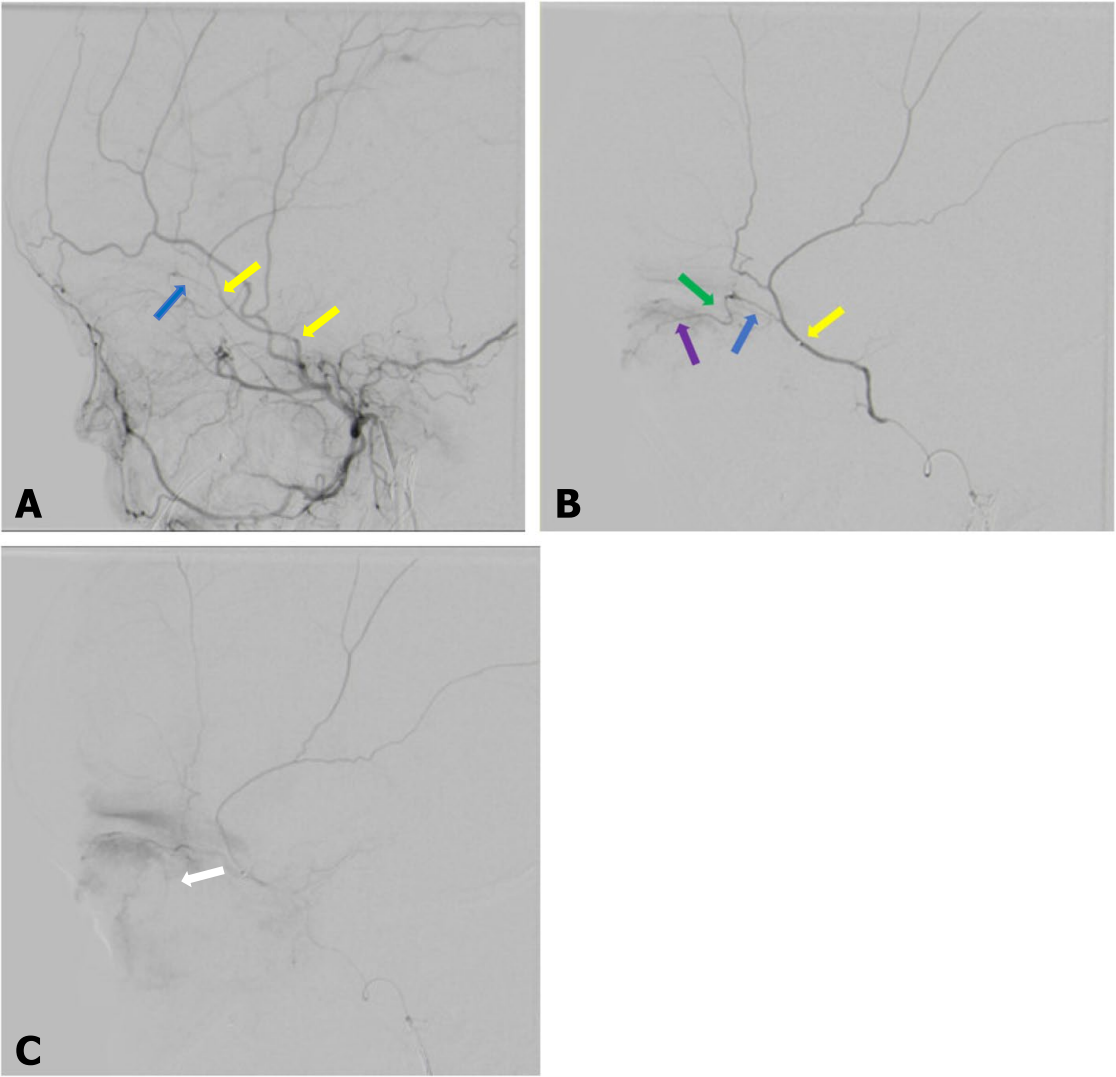
**A**, **B** and **C** Digital subtraction angiography of the right external carotid artery suggestive of meningo-ophthalmic variant (2A) confirmed by select injection of the right middle meningeal artery (2B) (Case 2). **A** The blue arrow illustrates the meningo-ophthalmic connection (middle meningeal artery to ophthalmic artery). The yellow arrow illustrates the selective injection of the middle meningeal artery. **B** The blue arrow illustrates the meningo-ophthalmic connection to the ophthalmic artery. The green arrow illustrates the ophthalmic artery. The purple arrow illustrates the central retinal artery with choroidal blush shown in the late phase (white arrow) (**C**)

A 2-year-old male Black South African patient was diagnosed with bilateral retinoblastoma at hospital. The left eye was enucleated, and the patient was not responding to systemic chemotherapy. A chemotherapy substance (melphalan) was planned to be injected directly into the OA to save the right eye. The patient underwent DSA, and the right ICA course displayed no OA filling. Tracking of the right ECA illustrated MMA filling the OA via meningo-ophthalmic variant, and the OA entered the orbit via the SOF (Fig. 2A and B). This was selectively accessed with a microcatheter, and intra-arterial melphalan was given. The left OA had a standard origin from the supraclinoid part of the ICA and coursed through the optic canal. The session was repeated after one month with a good clinical response.

## Discussion and conclusion

The development of the OA remains controversial due to inconsistent reports and views in the literature. Generally, embryogenesis involves multiple anastomoses, regression, and persistence of numerous primitive vessels, depending on the developmental stage. Some authors have hypothesized that adult OA develops from the persistence of primitive ventral OA while the primitive dorsal OA regresses [5, 9, 10]. This theory may be partially due to the course of the ventral OA through the optic canal while the dorsal OA penetrates the orbit through the superior orbital fissure [10]. In a recent report, Bracard and co-authors premised that adult OA is formed from the anastomosis between the primitive dorsal and ventral OA [11]. Other authors have suggested that the adult OA develops from a collateral connection between the two primitive OA and some adjacent vessels, including the MMA, at the 16–18 mm developmental stage [6, 8]. Notably, these authors agreed that dorsal and ventral OA is present at early gestation (4–9 mm stage) before the formation of adult OA. Variation in origin of the OA from the MMA as reported in our patients (Case 1 and 2) may be due to the persistence of interconnection between the primitive OAs and MMA during embryogenesis after migration of MMA as a branch of ECA [8].

The true incidence of variation in the origin of the OA is not clear, as most of the available reports are from international populations (Asian, American, and European). Most of the studies on prevalence are from the Japanese population, with no previous report from the African continent [4, 5]. Uchino and co-authors, in a large series study, reported a prevalence of 1.45% of OA arising from the MMA in a Japanese population using magnetic resonance angiographic images [5]. Liu et al. reported a similar case in an American population using a cadaveric subject [1]. In a recent report on France's population, Bracard et al. reported a case of OA duplication with both limbs arising from different segments on the ICA and coursing differently through the optic canal and superior orbital fissure [11]. OA may have a duplicate origin with contributions from MMA and ICA [12]. Variation in the origin of the OA has been associated with variation in its course. OA may enter the orbit through the SOF, an atypical foramen in the optic strut, or through the meningolacrimal foramen [13] instead of the optic canal. The most reported is the course of the OA through the SOF [11, 12]. Similar to the present cases, the two OA have atypical courses through the SOF (Figs. 1 and 2). Some authors have hypothesized the possibility of right-sided predominance, but there is no report on gender dominance [5]. Other researchers using similar sample size and population (Japanese) have reported that the incidence of variation in origin had no laterality/side dominance [4]. Although the two cases in the present study were on the right side, it is not enough to make a concluding statement on laterality prevalence. Variation in origin of the OA has been associated with the site of origin of the central retinal artery. The central retinal artery can arise from the ICA instead of the OA when there is variation in origin [12]. In the present study, central retinal artery arose from the OA of MMA origin (Fig. 2B). Histological analysis has shown that collagen content is significantly reduced in vessels with variant anatomy which may be a cause of mechanical weakness that could predispose the artery to vascular malformations such as aneurysms [5]. Some authors have hypothesized that the weakness in the arterial wall may be due to non-migration of the ventral OA inferiorly during embryogenesis or failure of the fusion of the primitive OA [4, 6].

Clinically, ligation or embolization of the MMA in treating conditions such as meningioma and aneurysm may result in visual complications or even post-operative blindness when an OA of variant origin from the MMA is involved [14, 15]. For instance, occlusion of the MMA in the presence of this variation may result in blindness [14]. Preoperative knowledge of this variant anatomy will inform the choice of treatments in interventions involving the OA and the central retinal artery that branch off it.

The authors describe a rare variation in the origin of the OA from the ECA branches, MMA in an adult Indian female and a pediatric Black male South African patient. It is important to identify the presence of variation in the origin and course of the OA prior to surgical intervention around the ICA and ECA, including their branches. Knowledge of this variation will help identify the possibility of variation when the OA is not found in its usual location. This information will inform the choice of intervention to carefully protect the variant artery from iatrogenic injury.

## Data Availability

Available on request. The corresponding author, Bukola Omotoso, can be contacted to request the data from this study.

## Abbreviations

OA: Ophthalmic artery
SOF: Superior orbital fissure
MMA: Middle meningeal artery
ICA: Internal carotid artery
ECA: External carotid artery
